# A Combined Ultrafiltration/Diafiltration Step Facilitates the Purification of Cyanovirin-N From Transgenic Tobacco Extracts

**DOI:** 10.3389/fbioe.2018.00206

**Published:** 2019-01-09

**Authors:** Patrick Opdensteinen, Juliana I. Clodt, Catherine R. Müschen, Volkan Filiz, Johannes F. Buyel

**Affiliations:** ^1^Fraunhofer Institute for Molecular Biology and Applied Ecology IME, Aachen, Germany; ^2^Institute for Molecular Biotechnology, RWTH Aachen University, Aachen, Germany; ^3^Institute of Polymer Research, Helmholtz-Zentrum Geesthacht, Geesthacht, Germany

**Keywords:** cyanovirin-N, host cell protein, particle size distribution, plant-derived biopharmaceuticals, protein purification, regenerated cellulose, RuBisCO, zeta potential

## Abstract

The production of biopharmaceutical proteins in plants offers many advantages over traditional expression platforms, including improved safety, greater scalability and lower upstream production costs. However, most products are retained within plant cells or the apoplastic space instead of being secreted into a liquid medium, so downstream processing necessarily involves tissue and cell disruption followed by the removal of abundant particles and host cell proteins (HCPs). We investigated whether ultrafiltration/diafiltration (UF/DF) can simplify the purification of the model recombinant protein cyanovirin-N (CVN), an ~ 11 kDa HIV-neutralizing lectin, from tobacco extracts prior to chromatography. We compared different membrane types and process conditions, and found that at pH 8.0 and 50 mS cm^−1^ an UF step using a 100 kDa regenerated cellulose membrane removed more than 80% of the ~ 0.75 mg mL^−1^ total soluble protein present in the clarified plant extract. We recovered ~ 70% of the CVN and the product purity increased ~ 3-fold in the permeate. The underlying effects of tobacco HCP retention during the UF/DF step were investigated by measuring the zeta potential and particle size distribution in the 2–10,000 nm range. Combined with a subsequent 10 kDa DF step, this approach simultaneously reduced the process volume, conditioned the process intermediate, and facilitated early, chromatography-free purification. Due to the generic, size-based nature of the method, it is likely to be compatible with most products smaller than ~50 kDa.

## Highlights

- Recombinant cyanovirin-N can be purified from clarified plant extracts by UF/DF.
- A regenerated cellulose membrane with a 100-kDa cut-off achieved efficient purification.
- Buffer pH and detergents influenced the zeta potential and particle size distribution of tobacco HCPs.
- UF/DF-based purification of recombinant proteins can simplify downstream processing.

## Introduction

Plants are advantageous over cell-based approaches for the production of biopharmaceutical proteins due to the inability of plants to support the replication of mammalian viruses, the greater scalability of whole plants compared to fermenters, and the lower capital and operating costs of upstream production (Tuse, 2011; Buyel et al., 2017). However, recombinant proteins produced in plants typically accumulate inside the plant cells and must be released by disruption, which also generates large amounts of particulates and abundant soluble host cell proteins (HCPs). Despite recent advances in the removal of such impurities (Buyel et al., 2015), downstream processing (DSP) in plant-based systems remains challenging due to the abundance of soluble HCPs in the clarified plant extract (Wilken and Nikolov, 2012; Buyel, 2015), especially if affinity purification steps such as Protein A chromatography are not available for product capture. Ribulose-1,5-bisphosphate carboxylase/oxygenase (RuBisCO) is the major HCP in tobacco, accounting for up to 29% of the total soluble protein (TSP). This is equivalent to ~0.9 g L^−1^ or 3.5 g kg^−1^ biomass based on a combined analysis using Bradford assay for total protein quantitation and densitometric analysis of LDS-PAA gels to obrain relative protein abundance (Buyel et al., 2013). RuBisCO and other HCPs can thus reduce the product-specific binding capacity during early capture steps (Buyel and Fischer, 2014d), increasing the costs for the corresponding equipment and media. Several methods have been developed to remove HCPs before, during or after extraction, including centrifugal extraction (Turpen, 1999), rhizosecretion (Drake et al., 2009), precipitation (Holler et al., 2007), pH shift (Hassan et al., 2008; Buyel and Fischer, 2014b), and heat treatment (Buyel et al., 2014a; Menzel et al., 2016), but these methods are not applicable to all products, for example due to thermal or pH sensitivity as observed for a malaria vaccine candidate expressed in *Nicotiana benthamiana* (Menzel et al., 2018).

In contrast, ultra-/diafiltration (UF/DF) is a gentle, size-based separation method that can also be used for the concentration and conditioning of process intermediates (Cromwell et al., 2006). Size-based purification is often prudent because many HCPs form multimers, e.g., a ~560 kDa hetero-hexadecamer in the case of RuBisCO (Buyel et al., 2015), which can thus be separated from smaller recombinant proteins. However, the separation performance is reduced by membrane fouling (Hadidi and Zydney, 2014), which involves the deposition of suspended or dissolved substances on the membrane surface or in its pores (Koros et al., 1996). Fouling can be prevented or minimized by the careful selection and adjustment of membrane properties and filtration conditions, such as pore size, hydrophilicity, transmembrane pressure, and pH (Koros et al., 1996; Dosmar, 2005; Cromwell et al., 2006).

**Figure 1:**
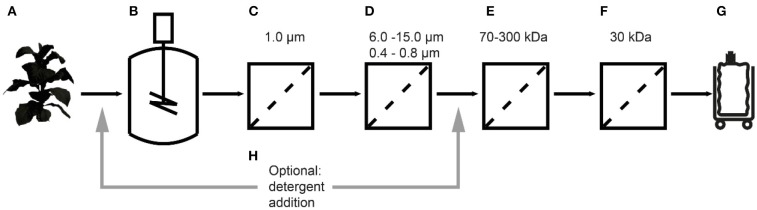
Extraction and filtration process flow for the purification of cyanovirin-N starting with transgenic plants **(A)**, extraction **(B)**, bag filtration **(C)**, depth filtration **(D)**, a first **(E)** and second **(F)**, ultra-/diafiltration step, and process intermediate **(G)**. The effect of detergent addition **(H)** at two process stages was investigated.

Here we report the purification of recombinant cyanovirin-N (CVN), an ~11 kDa HIV-neutralizing lectin, from *Nicotiana tabacum* (tobacco) extracts using different UF/DF membranes in a design-of-experiments (DoE) approach (Figure 1). The effect of pore sizes, separation conditions and additives on product purity and recovery were evaluated using CVN as a model recombinant protein. The effect of integrating UF/DF is discussed in terms of overall process economics.

## Materials and Methods

### Plant Cultivation and Extraction

Transgenic tobacco (*Nicotiana tabacum* cv. Petit Havana SR1) variety expressing CVN was cultivated as previously described (Buyel and Fischer, 2012) and harvested after 52–60 days. The plant material was stored at −20°C and extraction was carried out as previously reported (Buyel et al., 2014b) using three volumes of extraction buffer (3 mL g^−1^ biomass) and the same pH and conductivity as in the subsequent UF/DF step. We used citric acid buffer for pH 4.0–5.5, phosphate for pH 7.0–8.0, and glycine for pH 9.0, as well as conductivities in the 15–100 mS cm^−1^ range (equivalent to ~125–1,325 mM sodium chloride). Additives were included before or after extraction in different concentrations (Table S1).

### Extract Clarification

Extracts were clarified using a BP-410 bag filter (Fuhr, Klein-Winternheim, Germany) and a double-layer PDH4 depth filter (Pall, Dreieich, Germany) as previously described (Buyel and Fischer, 2014a), and in selected cases were also filtered using an Emphaze AEX Hybrid purifier (3 M, Neuss, Germany). The extracts and filtrates were monitored for turbidity, pH and conductivity, and were passed through a Satopore Capsule 0.20-μm filter (Sartorius-Stedim Systems GmbH, Göttingen, Germany) before UF/DF.

### Ultrafiltration/diafiltration

A Sartocon Slice 200 bench-top system (Sartorius) was used for all UF/DF experiments with a transmembrane pressure of 1.1 bar and a flow rate of 250 mL min^−1^ if not required otherwise by the DoE setup. Standard regenerated cellulose (RC) and polyether sulfone (PESU) membranes (Sartorius) with a 200 cm2 filter area were fed with 200 mL of clarified plant extract. Our custom membranes with a pore size of 8.5–100 nm and a 17 cm2 surface area (Rangou et al., 2014) were made from polystyrene-block-poly(4-vinylpyridine) (PS-*b*-P4VP) isoporous diblock copolymer (PSBC), polyacrylonitrile (PAN) or polyvinylidene difluoride (PVDF) with 50% (m/m) titan dioxide and fed with 50 mL of the extract. By default, four cycles of 4-fold feed concentration were conducted per run. After each concentration cycle, buffer with the same pH and conductivity as the feed was added to restore the feed starting volume. If not mentioned otherwise (e.g., in a DoE context) a pH of 7.5 and a conductivity of 50 mS cm^−1^ was used. The membrane molecular weight cut-off (MWCO) values were transformed to pore sizes using Equation 1 (Erickson, 2009; Zhang et al., 2018).

(1) $$\begin{array}{r} \text{Pore size }\left[\text{nm}\right]\;=\;0.09\times {\left(MWCO\left[Da\right]\right)}^{0.44} \end{array}$$

### Regeneration of UF/DF Membranes

UF/DF membranes were washed with (i) extraction buffer, (ii) 1 M sodium hydroxide, and (iii) ultra-pure water before storage in 20% [v/v] aqueous ethanol. The ultra-pure water wash was carried out using 20 L wash volume per m^2^ membrane area whereas 10 L m^−2^ was used in all other steps. The normalized water permeability (NWP) values of the membrane before and after use, and after regeneration, were calculated for runs with ultra-pure water using Equation 2 (Nestola et al., 2014). We used a temperature correction factor of 1.072 at 22°C as provided by the manufacturer, which differed slightly from published values (Kestin et al., 1978).

(2) $$\begin{array}{r} \text{NWP}\left[\frac{\text{L}}{\text{s }\cdot {\text{m}}^{2}\cdot \text{ Pa}}\right]=\;\frac{\text{PF }\cdot \text{ TCF}}{\text{TMP }\cdot \text{ membrane area }} \end{array}$$

where PF, permeate flow [g s^−1^]; TCF, temperature correction factor [–]; and TMP, transmembrane pressure [Pa].

Washing and data recording for the calculation of NWP values was conducted at a flow rate of 250 mL min^−1^ (inlet pressure = 0.7 bar) and a transmembrane pressure of 0.3 bar at 22°C.

Data were recorded with Satorius WinWedge software (SartoWedge PC interface software) and the corresponding Microsoft Excel worksheet.

### Gel Electrophoresis and Protein Detection

Process samples were characterized by lithium dodecylsulfate polyacrylamide gel electrophoresis and subsequent protein staining with Simply Safe-Stain or western blotting as previously described (Buyel and Fischer, 2014c). CVN was detected using the primary rabbit-anti-CVN polyclonal antibody 300i072BαCVN at a concentration of 0.08 μg mL^−1^ and a secondary goat-anti-rabbit antibody labeled with alkaline phosphatase (Jackson Immuno Research Laboratories, West Grove, USA) diluted 1:5,000 in 5% [m/v] milk powder in PBS-T.

The CVN band intensity was used as a surrogate for product concentrations and was determined by densitometry using ImageJ software (National Institutes of Health, Maryland, USA). Scanned western blot images were transformed to 16-bit grayscale, black-white inverted and the band intensity was quantified. The TSP was determined using the Bradford assay as described (Buyel and Fischer, 2014b). Native PAGE was conducted using 4–16% gradient gels according to the manufacturer's protocol (Thermo Fisher Scientific, Waltham, United States).

### UF/DF Performance Evaluation

TSP recovery in the UF/DF permeate was calculated using Equation 3.

(3) $$\begin{array}{r} \text{TSP}\left[\%\text{ initial}\right]\;=\;\frac{\sum_{1}^{n}\left(P_{i}\times V_{Pi}\right)}{\left(\text{F }\times \;{\text{V}}_{\text{F}}\right)}\;\times \;100\% \end{array}$$

where P_i_, protein concentration in the ith permeate [g L^−1^]; F, protein concentration in feed [g L^−1^]; V_Pi_, volume of the ith permeate [L]; and V_F_, feed volume [L]. Here, i = 1, 2, 3, 4.

The recovery of CVN after UF/DF was calculated as the fraction of CVN mass in permeate and feed represented by the densiometric CVN signal derived from the western blots of the corresponding samples. CVN purity was calculated as the ratio of the CVN signal derived from the western blots to the TSP value derived from the Bradford assay. The purity increase after UF/DF was calculated as the ratio of the CVN purity in the permeate and feed.

### Design of Experiments

Design Expert 8.0 was used to set up and analyze IV-optimal designs consisting of 16–18 runs as described before (Buyel and Fischer, 2014e). The factors were pH (4.0, 4.5, 6.25. 7.0, 8.0, and 9.0), conductivity (15, 50, and 100 mS cm^−1^), detergent concentration (0, 10, 50, and 90% critical micellar concentration, CMC), and detergent type (coded as the positive and negative charge (−1, 0, +1) of the corresponding detergent molecules (Table S1).

## Results and Discussion

### Regenerated Cellulose Membranes Suffer Less Severe In-process Fouling than Polyether Sulfone Membranes

**Figure 2:**
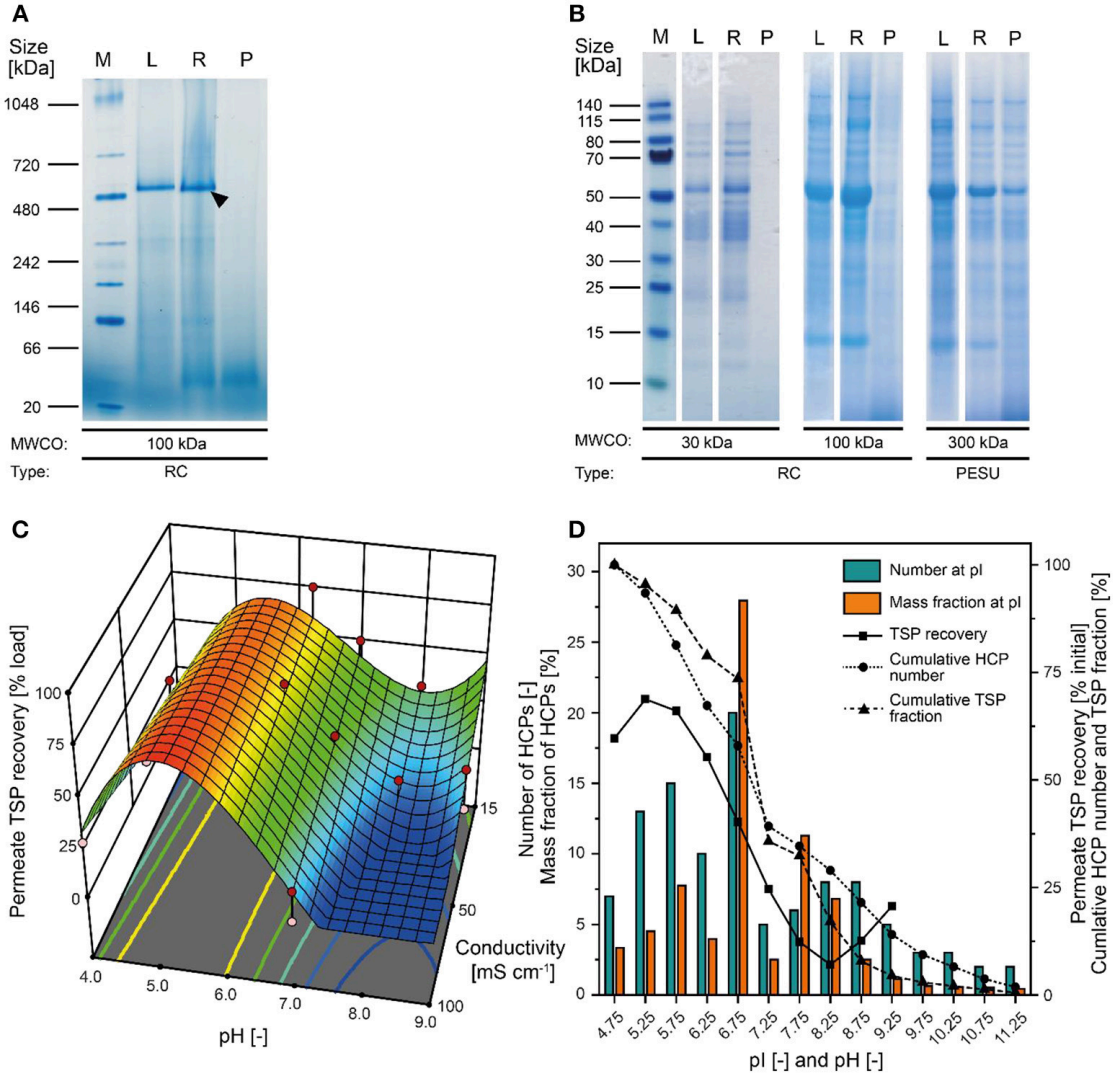
Separation of tobacco HCPs by UF/DF. **(A)** Native PAGE of clarified tobacco extract and UF/DF samples using a 100 kDa RC membrane (pH 7.6, 50 mS cm^−1^). The black arrow indicates RuBisCO (~560 kDa). **(B)** LDS-PAGE analysis of the protein composition of UF/DF samples (pH 7.0 and 50 mS cm^−1^) after separation using different membranes. **(C)** Response surface of TSP recovery in the UF/DF permeate of a 100 kDa RC membrane relative to the load, showing dependence on pH and conductivity. **(D)** The pI-dependent distribution of tobacco HCPs and the corresponding mass fractions in plant extracts (Buyel et al., 2013). The bin width was 0.5. The permeate TSP recoveries were predicted by the model displayed in **(C)** for 50 mS cm^−1^. MWCO, molecular weight cut-off; PESU, polyether sulfone; RC, regenerated cellulose; PSBC, polystyrene block copolymer; L, load; R, retentate; P, permeate.

Our analysis of tobacco extracts revealed that the major plant HCP RuBisCO formed oligomers larger than 480 kDa even after extraction (Figure 2A). We therefore concluded that size-based separation from recombinant proteins such as the ~11 kDa lectin CVN should be feasible by UF/DF, as previously speculated (Buyel et al., 2015). Therefore, we investigated different membrane materials, molecular weight cut-off (MWCO) ratings and process conditions, first in terms of the NWP to assess the compatibility of the membrane materials with plant-derived feed streams. We found that hydrophilic RC membranes with a MWCO of 100 kDa (300 kDa RC not available) had a significantly higher NWP before (~25%) and after regeneration (~16%) than the widely-used PESU membranes (100 and 300 kDa MWCO) despite the larger pore size of the latter (Figure S1). We concluded that RC was less susceptible to membrane fouling than PESU under our process conditions, which is favorable because it ensures a stable permeate flux and reproducible conditions between experiments, and reduces operational costs arising from the need for extensive membrane cleaning (Sommerfeld and Strube, 2005). Our results support previous studies in which RC also outperformed PESU and other synthetic membranes (Amanda and Mallapragada, 2001; Susanto et al., 2007). We also found that TSP recovery in the permeate was higher for the 100 kDa RC membrane than the PESU counterpart with the same MWCO (Table 1). This may reflect subtle pore size variations between the membranes, or may indicate a concentration polarization effect that can prevent effective passage of molecules through a PESU membrane (van den Berg and Smolders, 1989). Also, membrane-protein interactions that cause fouling may be responsible for the differences between the membranes. These interactions can be driven by either hydrophobic forces resulting from conformational changes of the proteins (Truskey et al., 1987) or by electrostatic attraction (Palecek and Zydney, 1994). Both mechanisms can potentially contribute to the differences in fouling we observed between RC, PESU PSBC membranes because the latter two are formed from polymers that contain aromatic groups that can interact with hydrophobic amino acids in proteins. Additionally, the apparent zeta-potential of RC membranes was about half of that of the PESU counterparts (Figure S3G) reducing the strength of potential electrostatic interactions with HCPs, half of which (mass wise) should be positively charged at our experimental pH of 7.5 according to their isoelectric point (Figure 2D).

**Table 1:** Properties and performances of different membranes in terms of CVN purification.

**ID**	**Material [–]**	**Membrane area [m^**2**^]**	**MWCO [kDa]^a^**	**Pore radius [nm]**	**Membrane thickness [μm]**	**Permeate CVN recovery [% initial]**	**Permeate TSP recovery [% initial]**	**CVN Purity increase [–]**	**Completed UFDF cycles [–]**
A	PSBC	1.7·10^−3^	~150	8.5	50	73.90	93.92	0.79	4
B	PSBC	1.7·10^−3^	~325	12.0	11	92.89	78.73	1.18	2^b^
C	PSBC	1.7·10^−3^	~1,200	21.0	41	66.20	68.43	0.97	2^b^
D	PSBC	1.7·10^−3^	~3,500	34.0	31	81.03	62.54	1.30	3^b^
E	PAN	1.7·10^−3^	~215	10.0	40	101.22	92.29	1.10	4
F	PVDF + 50% TiO_2_	1.7·10^−3^	~40,000	100.0	40	52.49	79.67	0.66	4
n.a.	RC	0.02	30	~4.2	180	n.a.	0.42	n.a.	3
n.a.	RC	0.02	100	~7.1	180	69.75	17.06	3.14	4
n.a.	PESU	0.02	100	~7.1	120	45.22	10.82	3.49	4
n.a.	PESU	0.02	300	~11.6	120	n.a.	97.90	n.a.	3

a MWCO was estimated based on Equation 1 for block copolymer materials.

b *The run was terminated due to the blocking of the membrane*.

Apart from the HCPs, plant extracts contain a vast number of other molecules, including DNA and cell wall fragments, carbohydrates and pigments. Colloids larger than 0.2 μm along with most pigments were removed during extract filtration using according adsorptive depth and membrane filters leaving only two populations of colloids of ~10 nm and ~150 nm (Figure S2C) in the clarified extract. Whereas, the former matched with the expected size of protein oligomers (2–10 nm), the identity of the latter colloid population remained unknown but based on its marginal volumetric fraction (Figure S2D) we deemed it unlikely to affect the UF/DF.

### Block Copolymer Membranes Rapidly Clog During the Processing of Clarified Tobacco Extracts

We also tested five novel polymer membrane materials (Table 1), but none of the combinations of pore size and membrane thickness achieved the selective retention of tobacco HCPs. Interestingly, even though the calculated MWCO rating of some of the membranes was close to or larger than that of the 300 kDa PESU membrane, only a few HCPs were observed in the corresponding permeates. As a result, the increase in CVN purity was a marginal ~1.3-fold (Table 1). Furthermore, membranes B, C, and D rapidly clogged during loading with the filtered tobacco extract. The brownish discoloration of these membranes (data not shown) indicated substantial fouling, which may explain the clogging and the unexpected retention of HCPs and CVN. Furthermore, the use of Equation 1 for the transformation of pore sizes into MWCO ratings may have been an oversimplification. Comparison with the RC and PESU membranes indicated that increasing the PSBC membrane thickness could potentially improve CVN purification, i.e., the PSBC membrane thickness was only about 25% of that of the other membranes (Table 1) and thickness can affect the selectivity of membranes (Kanani et al., 2010). In any case, the tested block copolymer membranes did not appear suitable for the purification of recombinant CVN from plant extracts. We therefore proceeded with a more detailed investigation of RC as the most promising membrane material.

### A 100 kDa MWCO Membrane Facilitates the Selective Retention of HCPs

**Figure 3:**
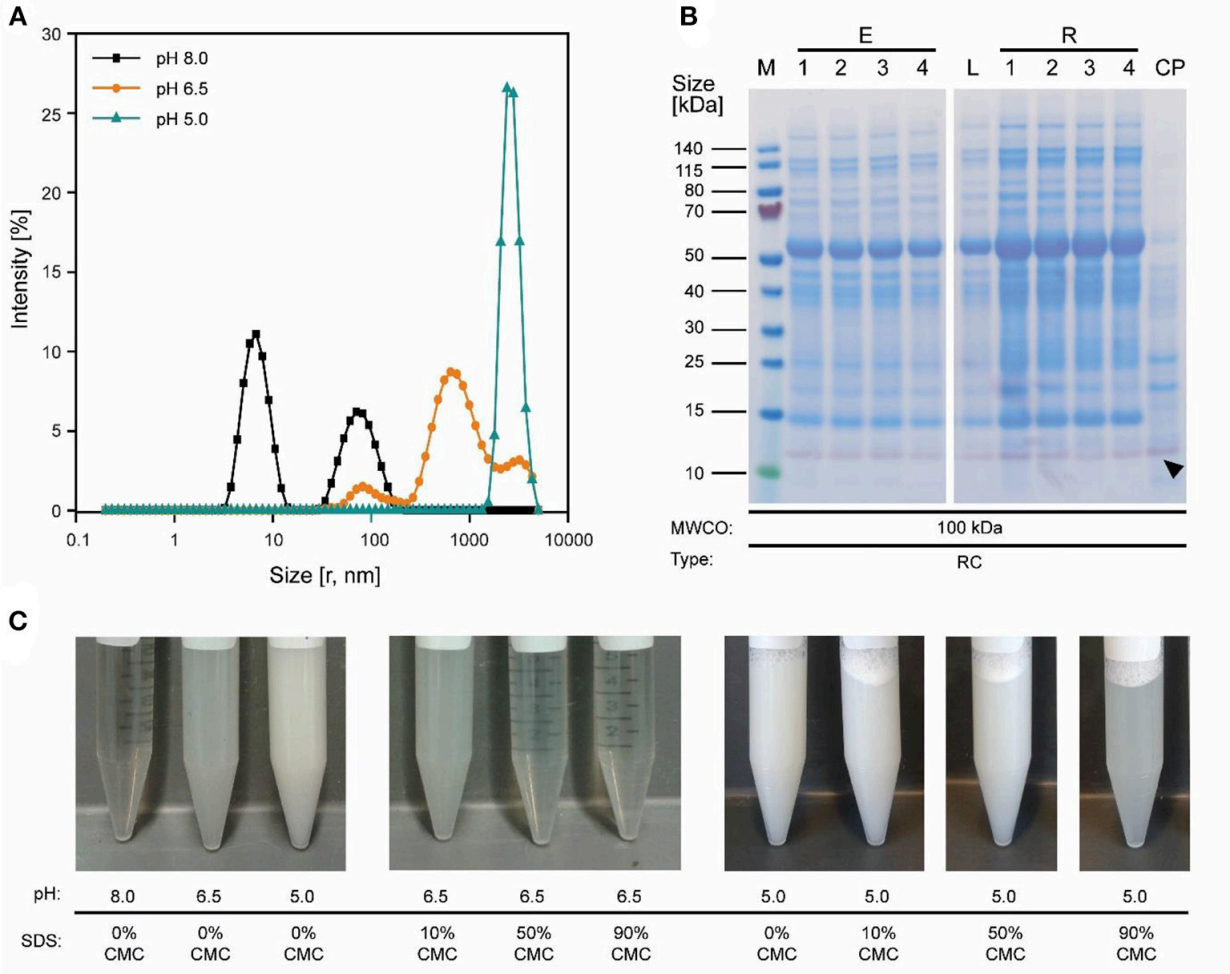
Effect of pH on protein aggregation and CVN purification using a 100 kDa RC membrane. **(A)** A shift in particle size distribution in clarified tobacco extracts occurred when reducing the pH from 8.0 to 5.0, as determined by dynamic light scattering. **(B)** Western blot (purple) and staining with Coomassie Brilliant Blue (blue) overlay of process samples from extraction to concentrated UF/DF-purified CVN (indicated by a black arrow). **(C)** The pH-sensitive formation of aggregates and the dispersion of said aggregates by adding the detergent SDS to the clarified tobacco extract. CMC, critical micellar concentration; CP, concentrated permeate; E1–E4, homogenate, conditioned extract, bag filtrate, depth filtrate; MWCO, molecular weight cut-off; r, calculated hydrodynamic particle radius; RC, regenerated cellulose; PSBC, polystyrene block copolymer; L, load; R1–R4, retentates after diafiltration steps 1–4; P, permeate.

Because the major tobacco HCP RuBisCO (apparent size, ~560 kDa) was retained by a 100 kDa membrane (Figure 2A), we next investigated RC membranes with MWCOs in the 30–100 kDa range as well as a 300 kDa PESU membrane (this MWCO rating was not available for RC membranes) to identify conditions suitable for the efficient separation of HCPs from CVN. Regardless of the conductivity (15–100 mS cm^−1^) and pH (pH 4.0–9.0), <1% (*n* = 3) of the TSP (including the product) passed through the 30 kDa membrane whereas close to all (98 ± 1%; ±SD, *n* = 2) passed through the 300 kDa membrane (Figure 2B). Only the 100 kDa membrane exhibited some degree of selectivity in terms of HCP retention. We therefore used a DoE approach to characterize HCP separation using this membrane in more detail, which yielded a predictive model of good quality (Figure 2C and Table S2). We found that the TSP recovery (excluding the product) in the permeate decreased from ~60 to 20% as the pH increased, which coincided with the distribution of the pI values of tobacco HCPs, i.e., high recovery at a pH close to the pI value representing many tobacco HCPs (Figure 2D). We speculated that this was the joined result of two effects. On the one hand, electrostatic protein-membrane interactions should be low close to the pI, thus increasing recovery, as previously reported (Fane et al., 1983; Burns and Zydney, 1999). This hypothesis was supported by our observation that the absolute zeta-potential of plant extract and purified RuBisCO decreased at lower pH values (Figure S2A and Table S3). Also, the zeta potential of the membranes decreased with decreasing pH (Figure S3G). On the other hand, the higher absolute TSP concentration in the feed at pH 8.0 (0.75 ± 0.17 g L^−1^; ±SD, *n* = 5) compared to pH 4.5 (0.14 ± 0.02 g L^−1^; ±SD, *n* = 2) may have caused stronger concentration polarization at high pH, further inhibiting recovery by preventing protein transport through the membrane (Jang et al., 2009). At pH 4.0, the absolute protein concentrations of 0.07 ± 0.05 g L^−1^ (±SD, *n* = 6) was close to the quantitation limit of the Bradford assay, probably limiting the predictive power of the DoE model. We assume that the low TSP concentrations at pH <5.0 reflected the protein aggregation we observed by dynamic light scattering, and the subsequent removal of these aggregates during clarification (Figure 3A). Also, by analyzing the UF/DF permeates with LDS-PAGE we found that for a pH of ~4 the fraction of proteins smaller than 20 kDa was substantially higher than at higher pH (Figure S3A–E). Therefore, the share of proteins able to pass the membranes was higher at low pH which likely caused the increased permeate recovery we observed under these conditions.

Conductivity had a relevant effect only at pH values above 7.0, where conductivities higher than 50 mS cm^−1^ reduced the TSP recovery in the permeate (Figure 2C). Even though salt can reduce the electrostatic exclusion of proteins from membrane pores and thus increase recovery (van Eijndhoven et al., 1995; Zeman et al., 1996), we assume that conductivities of ~100 mS cm^−1^ (~1.5 M sodium chloride) triggered protein adsorption to the membranes via hydrophobic interactions as described before (Fane et al., 1983).

### Conditions Supporting High CVN Recovery and High CVN Purity Do Not Overlap

The highest CVN recovery of 89 ± 5% (±SD, *n* = 2, Table 2) was observed at pH 4.5 and 50 mS cm^−1^, and was thus close to the theoretical pI of CVN (~5.0), a phenomenon observed for other proteins before, such as monomers and dimers of bovine serum albumin (BSA), and the human DNase dornase alfa (Fane et al., 1983; Burns and Zydney, 1999). In contrast, the greatest increase in CVN purity (3.14 ± 0.42, SD, *n* = 3) was achieved at pH 8.0 and 50 mS cm^−1^, but the CVN recovery was only ~70% (Table 2, Figure 3B). Interestingly, conditions supporting the greatest increase in CVN purity were associated with the highest absolute zeta potentials (Figure S2A, Table S3). We speculated that increasing the zeta potential artificially could therefore improve the purity of CVN even further. The zeta potential describes the electric potential of a moving, dispersed colloid (Hunter, 2013), and is thus affected by molecules binding to the colloid surface. We therefore tested several substances (Table S1) including various detergents known to interact with proteins or to improve UF/DF performance, in order to determine their effect on CVN purification (Jang et al., 2009). Because detergents such as SDS can interfere with protein quantitation methods such as the Bradford assay, we used appropriate controls to compensate for any offsets.

**Table 2:** Recovery and increase in purity of CVN in UF/DF permeates using a 100 kDa RC membrane. Standard deviations result from two or three runs.

**pH [–]**	**Conductivity [mS cm^**−1**^]**	**TSP recovery [% load]**	**CVN recovery [% load]^a^**	**CVN purity increase [–]^a^**	**Number of runs [–]**	**Zeta potential tobacco extract, monomodal analysis mode [mV ]**
4.50	50	63.02 ± 8.22	89.10 ± 4.70	1.43 ± 0.26	2	−4.27 ± 0.67 (pH 5.0, 7.57 mS cm^−1^)
6.25	50	46.80 ± 4.43	77.88 ± 5.03	1.68 ± 0.27	2	−9.94 ± 0.57 (pH 6.5, 6.53 mS cm^−1^)
8.00	50	22.30 ± 2.02	69.75 ± 6.68	3.14 ± 0.42	3	−13.07 ± 0.80 (pH 8.0, 6.32 mS cm^−1^)
6.25	15	47.22	67.49	1.42	1	n.a.
8.00	15	21.74	42.95	1.98	1	n.a.

a *Values ± standard deviation*.

### Detergents Can Increase the Purity but Not the Recovery of CVN During UF/DF

The simplest way to add detergents to our process was to include them in the extraction buffer. This increased the purity by 3.0 ± 0.6 (±SD, *n* = 2) and was thus comparable to the detergent-free approach. However, the CVN recovery in the UF/DF permeate was reduced to 42 ± 10% (±SD, *n* = 3) which was 30% lower than without detergents. In addition, the intense green color of the UF/DF feed indicated the presence of plant pigments (Figure S3F) which can interfere with protein purification, e.g., through covalent binding to the product (Barros et al., 2011; Wilken and Nikolov, 2012).

**Figure 4:**
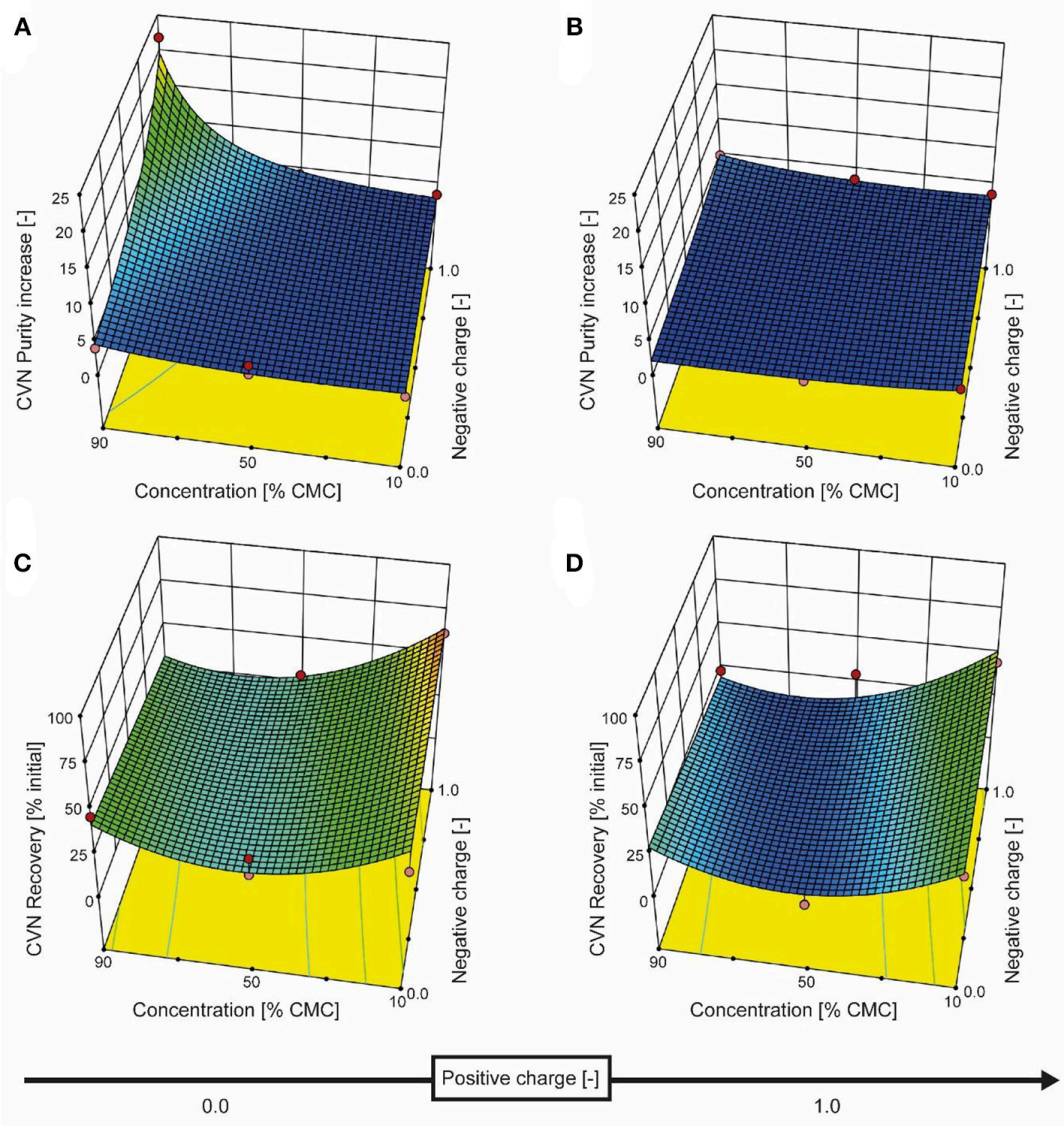
Response surface for CVN recovery and purity based on detergent charge and concentration. The detergent charge was coded as zero (0, charge type not present) or 1.0 (charge type present) for negative and positive charges, with the zwitterionic detergent having a charge coding of [1.0, 1.0]. The model for CVN recovery is depicted in the bottom row of panels (**C** and **D**), and the purity model is shown in the top row (**A** and **B**). Red dots represent actual experiments.

We therefore added the detergent after clarification. When we adjusted the pH of the clarified tobacco extract to 6.5 and 5.0 (starting from 8.0), the opacity of the liquid increased within seconds (Figure 3C) which we attributed to protein aggregation. However, when we subsequently added increasing amounts of SDS, the opacity gradually reduced, especially at pH 6.5 where the liquid became transparent again. Dynamic light scattering confirmed that the particle size distribution after the addition of SDS had a profile similar to that before the pH shift, indicating the presence of proteins (2–10 nm) and small particles (~80 nm) (Figures S2C,D). We used a DoE approach to characterize the effect of the detergent concentration and charge, which resulted in a model with good predictive quality (Figure 4 and Tables S4, S5). The highest CVN purity increase was ~20-fold using SDS at 90% CMC, which was seven times higher than without detergents. However, the CVN recovery was only ~22% instead of 70% in a detergent-free setup. The highest CVN recovery in the presence of detergents was 62% when the negatively charged detergent SDS was added at 10% CMC (Figure 4C), which was slightly less than the ~70% observed for the detergent-free setup. The increase in purity under these conditions was only ~3.4-fold, which was comparable to that of the detergent-free setup (~3.1-fold).

Adding the zwitterionic detergent Empigen-BB (90% CMC) or the non-ionic detergent Triton X-100 (90% CMC) increased the purity of CVN about 5- and 4-fold, respectively, whereas the cationic detergent CTAB did not improve CVN recovery or purity (Figure 4).

The addition of detergents reduced the average protein size in clarified extracts as determined by dynamic light scattering compared to the pH 8.0 standard (native size) from 7.84 ± 3.66 (mode ± standard deviation; *n* = 3 with >12 analytical replicates each; applies to all following sizes) to 5.85 ± 2.66 nm (Figures S2C,D). Our interpretation was that these results indicated the partial disassembly of oligomeric HCPs, which we confirmed for purified RuBisCO (native = 6.77 ± 2.37 nm, 90% CMC SDS = 4.36 ± 1.29 nm; pH 8.0, Figure S2B) and which was in agreement with previous reports (Jang et al., 2009). In the feed, this can increase the effective quantity of colloids that block membrane pores, which in turn can increase concentration polarization during UF/DF, allowing only small proteins such as CVN to pass through the membrane. Electrostatic repulsion between the membrane and charged HCPs (due to discord between the pH and pI, or interaction with the detergents) is also possible, because the charged detergent SDS increased the absolute zeta potential (Table S3) and was associated with higher purity as we speculated. In contrast, a pore-narrowing effect caused by the adsorption of more protein on the pore walls of the ultrafiltration membrane as the quantity of colloids increased seemed less likely, because this phenomenon is prevented by detergents (Brink et al., 1993).

### Anti-foaming Agents and Phenolic Impurities Do Not Affect CVN Recovery or the Increase in CVN Purity

We also tested whether the addition of polyvinylpolypyrrolidone (PVPP) could improve CVN recovery or purity because it is known to remove phenolic substances and high-molecular-mass compounds from plant extracts that may interfere with UF/DF (Loomis, 1974). In this context, we also tested the anti-foaming agent Polaxamer 188 (Pluronic F68), which can affect protein permeation during UF/DF (Kloosterman et al., 1988). When Polaxamer 188 was added prior to UF/DF, CVN recovery was 33.94 ± 0.74% (SD, *n* = 2) and the increase in purity was 3.4 and 2.4 at 10 and 90% CMC, respectively, thus offering no improvement compared to an additive-free setup. Adding PVPP before UF/DF did not affect the TSP (97 ± 6% initial, ±SD, *n* = 3) or the CVN recovery (~55%) or the increase in purity (2-fold). Removing phenolic compounds and DNA using a specialized Emphaze filter also had no effect on TSP (103%) or CVN recovery (55%) or the increase in purity (3.15-fold). We concluded that, in our process, phenolic compounds and DNA did not have a relevant effect on UF/DF performance using a 100 kDa RC membrane.

### Economic Relevance of UF/DF Purification Steps

Evaluating the economics of UF/DF is dependent on the process and product. In the case of plant-derived biopharmaceutical proteins for which no affinity purification step is available, UF/DF showed three clear advantages: (i) the large and expensive columns required for HCP binding (Buyel and Fischer, 2014b) can be avoided, (ii) processing times can be reduced, e.g., for 600 L plant extract from ~12 h in the case of a packed-bed column (5-L column, 30 cm bed height, 300 cm h^−1^ linear flow rate) to ~1 h (UF/DF with 2 m^2^ filter area, 1.25 bar transmembrane pressure), and (iii) the conditioning of process intermediates can be combined with concentration (Lightfoot et al., 2008), allowing the use of smaller and thus less expensive equipment due to the smaller volume streams. Based on the current CVN recoveries and purities (Figure 4), a detailed analysis of a hypothetical three-stage purification process (Table S6) revealed that incorporating UF/DF before the first chromatographic purification step can reduce the duration of DSP from 14 to 9 h with cost savings of about 15%. For this calculation, we assumed that UF/DF will increase the CVN recovery of the first chromatographic step from 0.5 to 0.7 due to the lower HCP burden. In the calculation, this allowed the omission of a third chromatography step, which was otherwise necessary to achieve a product purity >95%, but will require experimental verification once a full process is set up for CVN. Additional benefits of the UF/DF setup include the cost-saving potential of the smaller-scale equipment and therefore the smaller process footprint, e.g., due to an increased product-specific binding capacity of chromatography columns. However, this was not taken into account for the cost calculation because the effect will strongly depend on the pH at which a plant-based process is operated, i.e., at high pH the benefits of UF/DF will be substantial because large quantities of HCPs can be removed from the process intermediate whereas under acidic conditions a pH shift alone can be sufficient to remove HCPs.

## Conclusions

Among the membranes we tested, regenerated cellulose (RC) was the most suitable material for the purification of CVN from clarified tobacco extracts due to low membrane fouling. Unfortunately, RC membranes are currently not available with MWCOs between 100 and 300 kDa, which could improve the separation of HCPs from plant-derived recombinant proteins smaller than 50 kDa.

In addition to the MWCO, more tobacco HCPs were retained at high pH, increasing the purity of CVN by about 3-fold (3.14 ± 0.42; ±SD, *n* = 3) using a 100 kDa RC membrane at pH 8.0 and 50 mS cm^−1^. Adding the negatively charged detergent SDS resulted in an additional >20-fold increase in the purity of CVN, but at the expense of product recovery, which fell from ~70 to 20%. Therefore, selection of the MWCO should be accompanied by a careful adjustment of the separation conditions, which will help to control and improve recombinant protein purification from plant extracts using UF/DF in the future. Additionally, UF/DF may help to save 10–15% in DSP costs with additional savings being possible due to the smaller volumes and hence the smaller footprint of DSP equipment.

## Author Contributions

PO conducted the UF/DF experiments and analyzed the process samples. JC cast the custom membranes. CM prepared the protein solutions and purified protein samples. VF designed the custom membranes. JB designed and analyzed the UF/DF experiments and performed the cost calculations.

### Conflict of Interest Statement

The authors declare that the research was conducted in the absence of any commercial or financial relationships that could be construed as a potential conflict of interest.

## Abbreviations

CIP: cleaning in place
CMC: critical micelle concentration
CP: concentrated permeate
CVN: cyanovirin-N
DoE: design of experiments
DSP: downstream processing
HCPs: host cell proteins
MWCO: molecular weight cut-off
NWP: normalized water permeability
pI: isoelectric point
PS-*b*-P4VP: polystyrene-*block*-poly(4-vinylpyridine)
PSBC: PS-*b*-P4VP-diblock copolymers
RC: regenerated cellulose
RuBisCO, ribulose-1: 5-bisphosphate carboxylase/oxygenase
SD: standard deviation
SDS: sodium dodecylsulfate
UF/DF: ultrafiltration/diafiltration
TSP: total soluble protein
USP: upstream production.
