# Supplementary material for: A Combined Ultrafiltration/Diafiltration Step Facilitates the Purification of Cyanovirin-N From Transgenic Tobacco Extracts
Source: Front Bioeng Biotechnol. 2019 Jan 9;6:206. doi: 10.3389/fbioe.2018.00206 (PMC6334625; doi:10.3389/fbioe.2018.00206)
Supplement: Supplementary file 1 [file Data_Sheet_1.PDF]

Supplementary materials

Table S1: Additives tested during the extraction and UF/DF steps for the purification of CVN.

Additive [-]	Brand name [-]	Type [-]	Charge [-]	Added before /after extraction	Concentration [g L^{-1}] (mM) Low	Concentration [g L^{-1}] (mM) High	[% CMC] Low	[% CMC] High
Cetrimonium bromide (CTAB)	n.a.	Detergent	Cationic	-/+	0.034 (0.092)	0.302 (0.828)	10	90
N,N-Dimethyl-N-dodecylglycine betaine	EMPIG EN-BB	Detergent	Zwitterionic	+/+	0.059 (0.210)	0.527 (1.890)	10	90
Octyl phenol ethoxylate	Triton X-100	Detergent	Nonionic	-/+	0.013 (0.020)	0.116 (0.180)	10	90
Prolaxamer 188	Pluronic F68	Tenside	Nonionic	-/+	0.033 (0.004)	0.301 (0.036)	10	90
Polyvinylpolypyrrolidone (PVPP)	Divergan	Stabilizer	Nonionic	-/+	0 (0)	5 (n.a.)	n.a.	n.a.
Sodium dodecyl sulfate (SDS)	n.a.	Detergent	Anionic	+/+	0.236 (0.820)	2.128 (7.380)	10	90

CMC – critical micellar concentration

Table S2 Model factors with a significant influence on TSP recovery in the UF/DF permeate identified by analysis of variance (ANOVA).

Source	Sum of squares	Degrees of freedom	F-value	p-value	Model coefficient
Model	4473.38	5	10.18	0.002	n.a.
A (pH)	3259.09	1	37.08	<0.001	-80.41
B (conductivity)	427.24	1	4.86	0.055[a]	-8.74
AB	1216.82	1	13.84	0.005	-24.22
A^2	1940.21	1	22.07	0.001	-29.98
A^3	2498.66	1	28.43	<0.001	78.73
Residual	791.07	9	n.a.	n.a.	n.a.
Lack of fit	498.61	6	0.85	0.605	n.a.
Pure error	292.46	3	n.a.	n.a.	n.a.

Evaluation parameter	Value
R^2	0.850
Adjusted R^2	0.766
Predicted R^2	0.447

[a]Term B was included in order to maintain the model hierarchy.

Table S3: Zeta potentials of tobacco extracts and purified RuBisCO. Values are the mean zeta potential and standard deviation from three (extract) or six (RuBisCO) technical replicates. The monomodal analysis mode (fast field reversal) applies a rapidly alternating electric field and is suitable for samples with a conductivity >5 mS cm^{-1}. The general purpose analysis mode applies a combination of fast field reversal and slow field reversal. Due to increased joule heating, this mode poses the risk of denaturing the sample at salt concentrations >150 mM.

Sample [-]	pH [-]	SDS conc. [% CMC]	Conductivity [mS cm^{-1}]	Zeta potential [mV]	
				Monomodal analysis mode	General purpose analysis mode
Extract	5.0	0.0	7.57	-4.27±0.67	-3.91±0.31
	5.0	90.0	6.31	-21.87±0.76	-20.3±0.75
	6.5	0.0	6.53	-9.94±0.57	-9.96±0.33
	6.5	10.0	6.60	-15.77±0.76	-16.13±0.32
	6.5	50.0	7.04	-17.13±0.92	-18.57±0.90
	6.5	90.0	6.66	-17.80±1.51	-18.10±1.27
	8.0	0.0	6.32	-13.07±0.80	-11.53±0.21
	8.0	10.0	6.67	-15.53±1.58	-14.43±1.36
	8.0	50.0	6.72	-19.67±0.90	-17.30±0.92
	8.0	90.0	6.65	-18.07±1.76	-20.10±0.92
RuBisCO	6.5	0.0	5.72	-12.77±1.08	-13.52±1.33
	6.5	10.0	5.32	-18.86±0.96	-19.27±0.83
	6.5	50.0	5.17	-22.00±1.24	-22.97±0.47
	6.5	90.0	5.30	-21.58±1.95	-26.22±4.23
	7.0	0.0	5.85	-13.87±1.08	-15.02±0.53
	7.0	10.0	5.27	-17.25±1.47	-19.26±1.63
	7.0	50.0	5.64	-21.68±1.70	-21.02±2.26
	7.0	90.0	5.41	-24.74±1.53	-24.83±0.97
	8.0	0.0	6.69	-16.78±0.75	-16.82±1.34
	8.0	10.0	7.26	-16.48±1.90	-17.44±1.21
	8.0	50.0	7.23	-22.32±1.24	-23.12±1.04
	8.0	90.0	7.25	-23.40±2.31	-25.62±3.05

CMC – critical micellar concentration; SDS – sodium dodecylsulfate

Table S4: Model factors with a significant influence on the increase in CVN purity in the UF/DF permeate identified by ANOVA.

Source	Sum of squares	Degrees of freedom	F-value	p-value	Model coefficient
Model	0.15	6	8.90	0.001	n.a.
A (concentration)	0.019	1	6.80	0.024	-0.052
B (negative charge)	0.041	1	14.52	0.003	-0.054
C (positive charge)	0.029	1	10.01	0.009	0.045
AB	0.029	1	10.14	0.009	-0.063
AC	0.027	1	9.61	0.010	0.061
A^2	0.013	1	4.64	0.054	-0.052
Residual	0.031	11	n.a.	n.a.	n.a.
Lack of fit	0.018	6	1.14	0.451	n.a.
Pure error	0.013	5	n.a.	n.a.	n.a.

Evaluation parameter	Value
R^2	0.829
Adjusted R^2	0.736
Predicted R^2	0.538

UF/DF – ultrafiltration/diafiltration

Table S5: Model factors with a significant influence on CVN recovery in the UF/DF permeate identified by ANOVA.

Source	Sum of squares	Degrees of freedom	F-value	p-value	Model coefficient
Model	6460.35	5	17.18	<0.001	n.a.
A (concentration)	761.25	1	10.12	0.008	-9.83
B (negative charge)	37.00	1	0.49	0.496[a]	1.62
C (positive charge)	703.13	1	9.35	0.010	-6.61
AB	427.37	1	5.68	0.035	-6.42
A^2	1997.94	1	26.56	<0.001	19.91
Residual	902.52	12	n.a.	n.a.	n.a.
Lack of fit	724.84	7	2.91	0.129	n.a.
Pure error	177.68	5	n.a.	n.a.	n.a.

Evaluation parameter	Value
R^2	0.877
Adjusted R^2	0.826
Predicted R^2	0.691

[a]Term B was included in order to maintain the model hierarchy. UF/DF – ultrafiltration/diafiltration

Table S6: Cost calculation for CVN purification with and without a UF/DF step.

Process step	Parameter	Unit	Process	
			Regular	UF/DF
Feed	Volume	[L]	600	
	Product quantity	[g]	1	
	Product purity	[-]	0.01	
UF/DF	Duration	[h]	0	2
	Filter area	[m^2]	0	2
	Concentration factor	[-]	1	15
	Equipment cost	[€]	0	5000
	Product recovery	[-]	1	0.5
	Product quantity	[g]	1	0.5
	Purification factor	[-]	1	20
	Product purity	[-]	0.01	0.17
Chromatography 1	Column volume	[L]	6	0.8
	Bed height	[m]	0.3	0.3
	Linear flow rate	[$m\ h^{-1}$]	3	3
	Chromatography load volume	[L]	600	40
	Chromatography duration	[h]	10	5
	Equipment cost	[€]	6000	800
	Product recovery	[-]	0.5	0.7
	Product quantity	[g]	0.5	0.35
	Purification factor	[-]	20	20
	Product purity	[-]	0.17	0.80
Chromatography 2	Column volume	[L]	0.6	0.08
	Bed height	[m]	0.3	0.3
	Linear flow rate	[$m\ h^{-1}$]	3	3
	Chromatography load volume	[L]	12	1.6
	Chromatography duration	[h]	2	2
	Equipment cost	[€]	600	80
	Product recovery	[-]	0.5	0.5
	Product quantity	[g]	0.25	0.175
	Purification factor	[-]	20	5
	Product purity	[-]	0.80	0.95
Chromatography 3	Column volume	[L]	0.06	0
	Bed height	[m]	0.3	0.3
	Linear flow rate	[$m\ h^{-1}$]	3	3
	Chromatography load volume	[L]	1.2	0.16
	Chromatography duration	[h]	2	0
	Equipment cost	[€]	60	0
	Product recovery	[-]	0.5	1
	Product quantity	[g]	0.125	0.175
	Purification factor	[-]	5	1
	Product purity	[-]	0.95	0.95
Summary	Total time	[h]	14	9
	Labor costs	[€]	420	270
	Equipment cost	[€]	6660	5880
	Total costs	[€]	7080	6150

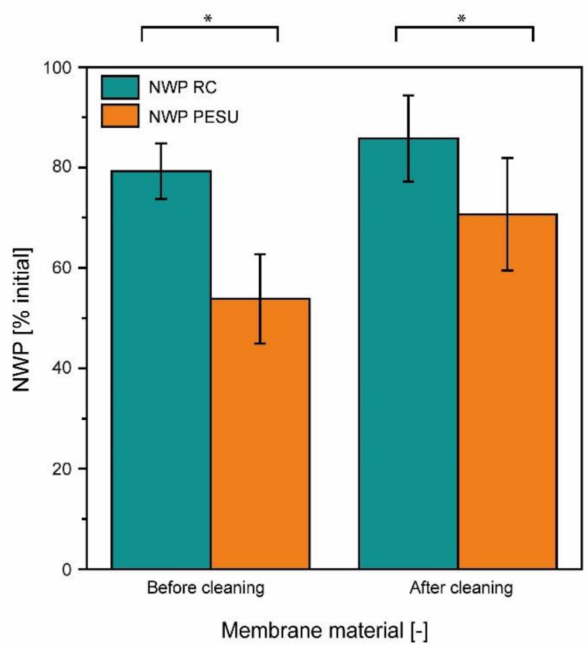

Figure S1: Fouling of RC and PESU membranes. The normalized water permeability (NWP) values for 100 kDa RC (300 kDa RC not available) as well as 100 and 300 kDa PESU membranes were calculated according to **Equation 2**. Error bars represent the standard deviation (n≥7). PESU – polyether sulfone; RC – regenerated cellulose. * denotes a statistically significant difference: two sample *t*-test (OriginPro 2015), significance level 5%, $p = 1.18741\times10^{-6}$ (prior to cleaning) and $p=0.00918$ (after cleaning).

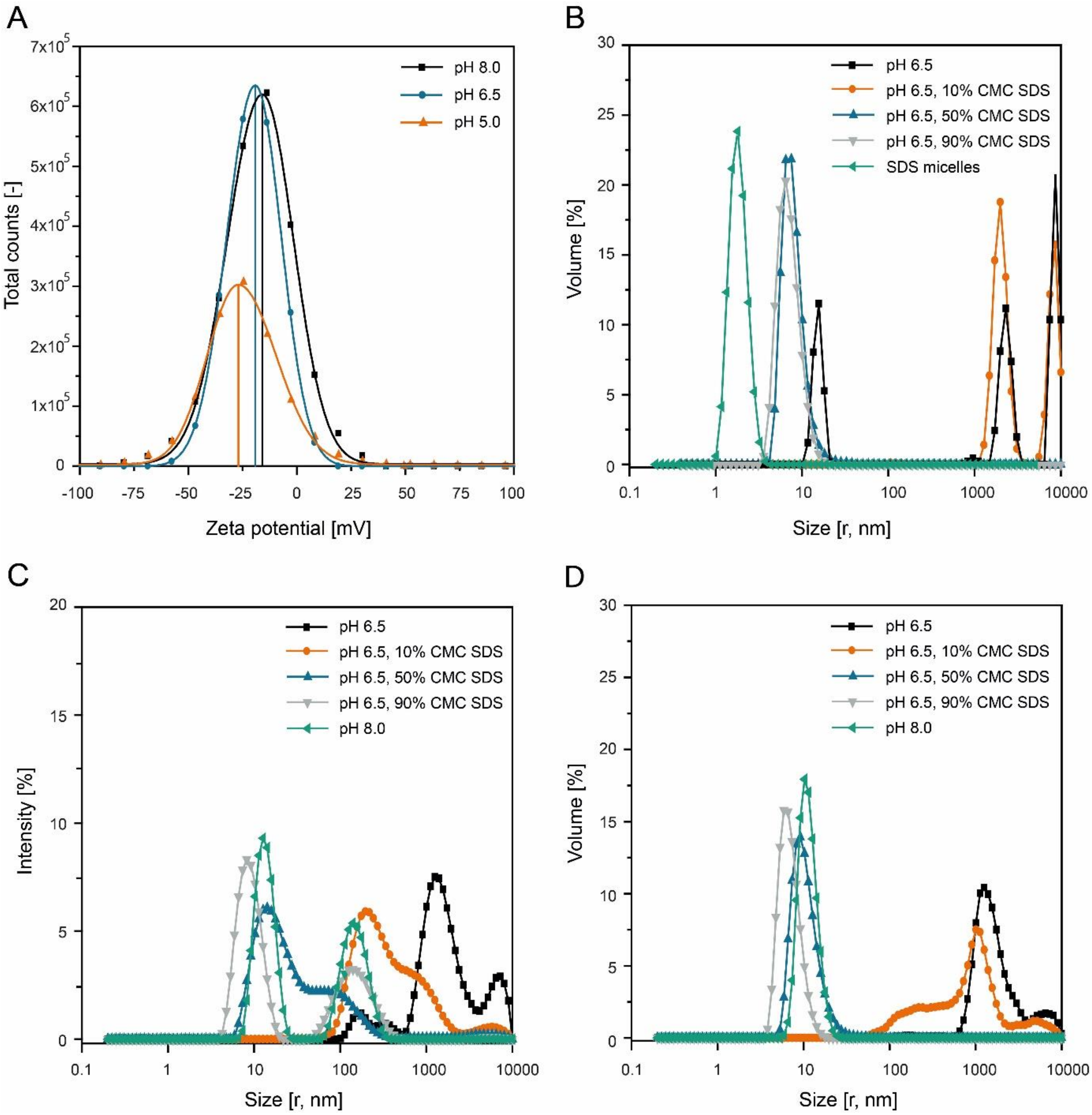

Figure S2: Particle size distributions and zeta potentials of purified RuBisCO and conditioned tobacco extract based on the pH and presence/absence of the detergent SDS. A. Zeta potential of purified RuBisCO with concentrations of 3.37 mg mL^{-1} at pH 6.5 and 7.0 and 1.69 mg mL^{-1} at pH 8.0. The conductivity was 5.54–6.48 mS cm^{-1}. B. Volume-based particle size distributions of purified RuBisCO (3.25 mg mL^{-1}) after a pH shift from 8.0 to 6.5. The conductivity was 5.13–5.38 mS cm^{-1}.C. Intensity-based particle size distribution of conditioned, 0.2-µm filtered tobacco extract (TSP = 5.48 mg mL^{-1} at pH 8.0, ~50 mS cm^{-1}). D. Volume-based size distributions for the same samples as in C. RuBisCO – Ribulose-1,5-bisphosphate carboxylase/oxygenase; SDS – Sodium dodecylsulfate, CMC – critical micellar concentration.

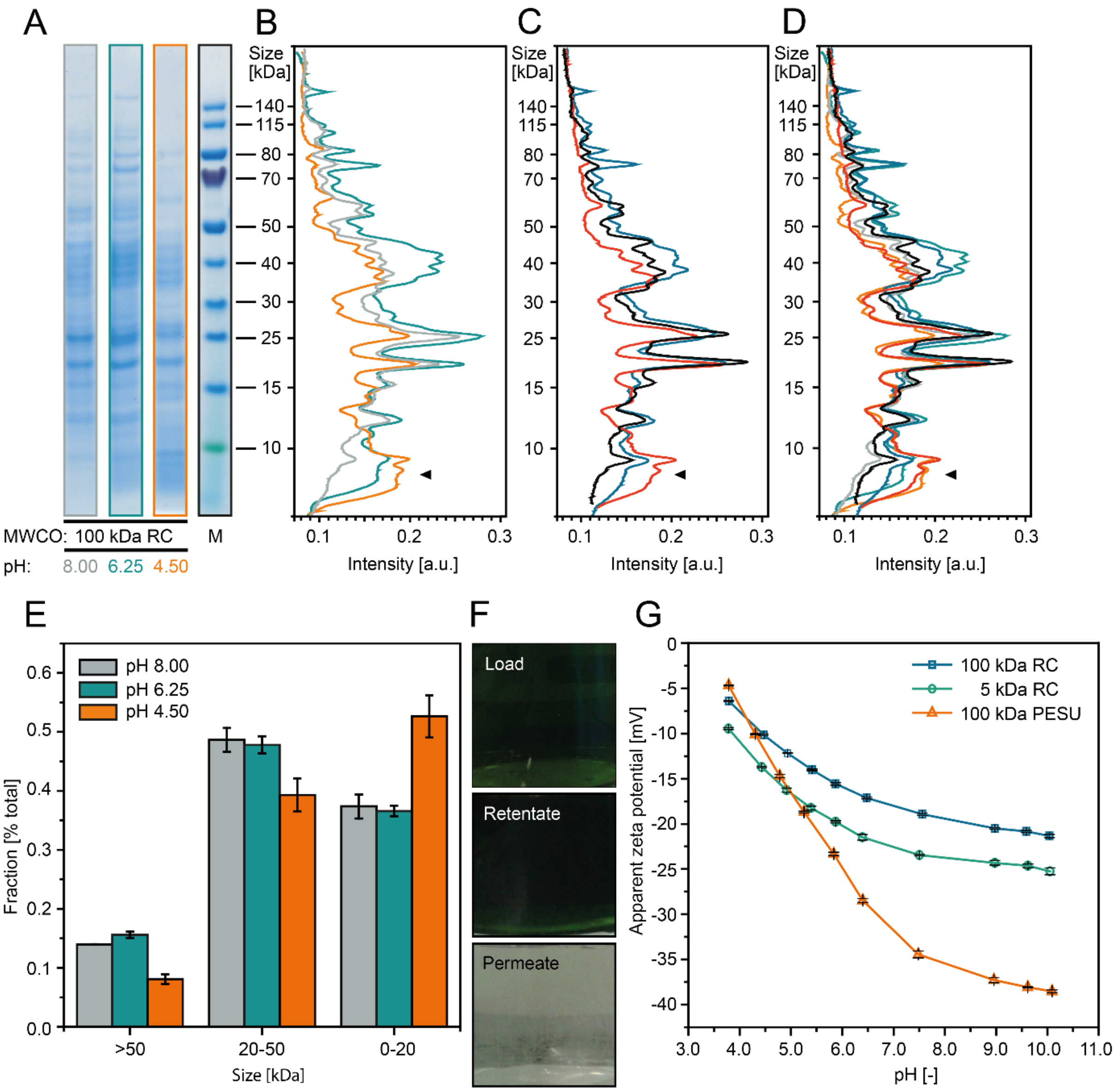

Figure S3: HCP analysis in UF/DF permeates at different pH values. A. Representative UF/DF permeate samples on Coomassie-staining of LDS-PAA gels. B. Densitograms of the samples from panel A. C. Results for biological replicates of samples from panel A/B. D. Overlay plot of densitograms from B and C. The black arrows indicate substantial differences between samples obtained at different pHs in the low protein size range. E. Clustering the fraction of the area under the curve of densitograms from panels B and C according to the protein size. Error bars indicate the standard deviation from two biological replicates. F. Color of UF/DF process intermediates after protein extraction in the presence of detergents. The load color without detergent can be seen in **Figure 2C** (pH 8.0, 0% CMC). G. Apparent

zeta potential of regenerated cellulose (RC) and polyether sulfone (PESU) membranes at different pHs.
